# AMN Congress 2022 – Report of the panel on minimizing the risks of failure in TBI research

**DOI:** 10.25122/jml-2022-1011

**Published:** 2022-07

**Authors:** Alexandra-Mihaela Gherman, Dafin Fior Muresanu, Andreea Strilciuc

**Affiliations:** 1. RoNeuro Institute for Neurological Research and Diagnostic, Cluj-Napoca, Romania; 2. Department of Neuroscience, Iuliu Hatieganu University of Medicine and Pharmacy, Cluj-Napoca, Romania

The theme question of the first panel of the 2022 AMN Congress – “Research in TBI is full of challenges – how to minimize the risks of failure?” was answered within a very dynamic and complex dialogue, coordinated by Prof. Dafin F. Muresanu, among the following panelists:

- Michael Chopp (USA);
- Karin Diserens (Switzerland);
- Andrew Maas (Belgium);
- Johannes Vester (Germany).

Michael Chopp (USA) provided the first answers using a dichotomy between pre-clinical and clinical trials, highlighting that it is extremely important to decide upon the appropriateness of the models [1] and sub-models (*e.g*., animal models or biomarkers) and the hypotheses to be tested (*e.g*., in TBI, what is happening both to the white matter and to the secondary developments after an initial injury to the brain) in the pre-clinical stage of the trial. Moreover, it is essential to know and understand the pre-clinical population of the patient [2] to establish a similar variety of populations of animals in complete clinical studies:

- Male;
- Female;
- Young;
- Old;
- Comorbidities, (*e.g*. Diabetes Mellitus, hypertension – which are critically important to any study).

Regarding the patient population in TBI and stroke, Michael Chopp draws attention to the fact that the start point should be the selection of the population in order to develop treatments for the pathophysiology of a neuronal injury to be understood. Then, within the selected (sub) population, it is necessary to have endpoints that would be relevant to the endpoints of interest in humans.

One example of an endpoint is a mechanism, *i.e*., in many models, it is sought to understand the therapeutic intervention, and how a specific therapy impacts a certain mechanism on average.

Going further with his explanation, Michael Chopp points to the fact that clinicians limit their orientation to the outcomes of a study and not to its mechanisms. For example, patients with TBI develop chronic, emotional, psychological, cognitive, and sleep-related problems in addition to deficits in motor functions.

In conclusion, pre-clinical trials are characterized by a 'weakness' which is further translated to the clinical studies into a need to refine and expand the outcomes measures in the pre-clinical study to be relevant to the doctors' interests when treating patients. The expectation would be to create a communication bridge between the pre-clinical and clinical world, having at one end the observations and questions from the clinical world and, at the other end, the controlled experiments performed by the pre-clinical world in order to illuminate the needs of the clinicians.

Andrew Maas agrees with the statements of the first panellist, also pointing out that, indeed, experimental research in TBI is focused on mechanisms, while the clinical studies are focused on outcomes. Based on that, two problems were easily identified:

- The lack of early mechanistic endpoints in the clinical stage – which is an issue that cannot be addressed because, for example, if there were both a drug that could act specifically on a mechanism and a target to measure that mechanism, then the world would be entirely different; indeed, there is a high interest in biomarkers, but these could only provide guidance, not create miracles;
- The patient is treated in the acute phase and then sent home and maybe consulted again after 6 months, the clinicians having no clue whatsoever about what happened to the patient in that interim period; in other words, if the treatment in the acute phase is not consolidated in the post-acute phase, all the benefits obtained in the acute phase are lost (even in high-income countries providing good post-acute care is an issue to be addressed).

Michael Chopp proposes a solution to the need for early mechanistic endpoints: to keep the animals/the patients under observation for an extended period and look at them systematically. Going further with his explanation, Michael Chopp states that in TBI and stroke, there are systemic organ effects that contribute to the patient's deficits. For this reason, when treating patients, one needs to be aware of what is done. For example, when treating a patient with head trauma, a whole set of very complicated and interactive effects are initiated that come together and synthesize into the status of the patient, *i.e*., emotional, cognitive, and motor deficits are all integrated effects of the primary injury, so the secondary injuries are present throughout the body (*e.g*., changes in the liver function, in the chemistry), and become systemic information that contributes to the patient's outcome. Andrew Maas sums everything up in a clear, short sentence, highlighting the fact that TBI is, in fact, a systemic disease, not only brain-oriented. To emphasize this conclusion, Michael Chopp adds that in TBI and in stroke, the inflammatory response is extremely important [3,4]; the vasculature in the brain becomes a source of toxicity and, according to his experiments, this results in a number of agents that do not just stay in the brain, but impact the heart, the liver, the kidney, and the feedback these organs provide. In other words, inflammation is extremely important because the initiating trauma becomes a risk factor for any systemic or cognitive dysfunction.

Karin Diserens also agrees that the pre-clinical and clinical worlds should come together to further develop studies and collect data, but she also points out another important risk of failure when it comes to research, namely the lack of equity between the studies developed and the reviewers. For example, as a lot of observational and individual assessment studies are developed in the field of TBI, reviewers also need to be educated to consider these studies and validate data without putting forward the idea that low evidence is not the truth, while high evidence is the truth [5]. Johannes Vester supports her statement, adding that observational studies are still considered to be low evidence, while various guidelines are taken as moderate evidence.

Nevertheless, from his point of view, different opinions lead to diversity which is also a language one can learn from; it is a reality reflecting different truths. Based on this, the important research risks can be controlled via observational and randomized trials in which heterogeneity can be solved with stratifications, with like-to-like comparisons.

***Basically, one needs to accept diversity in terms of evidence in order to find different things*.**
